# Minimally Invasive Reconstruction of Traumatic Segmental Femoral Bone Loss Using a Non-Vascularised Ipsilateral Fibular Graft in a Three-Year-Old Child: A Case Report

**DOI:** 10.7759/cureus.109818

**Published:** 2026-05-28

**Authors:** Tashi G Khonglah, Chirag Sharma, Sharat Agarwal, Bhaskar Borgohain, Penthungo Ezung

**Affiliations:** 1 Orthopaedics and Traumatology, North Eastern Indira Gandhi Regional Institute of Health and Medical Sciences, Shillong, IND

**Keywords:** bone remodelling, femoral bone loss, fibular graft, limb salvage, paediatric trauma

## Abstract

Traumatic segmental bone loss of the femur in very young children is exceedingly rare and presents significant reconstructive challenges. Although several reconstructive options, including distraction osteogenesis, vascularised bone grafting, and the induced membrane technique, have been described, each carries inherent limitations, particularly in the paediatric population. We report the case of a three-year-old child who sustained traumatic extrusion of a segment of the femoral shaft following a motor vehicle accident. Reconstruction was performed using a minimally invasive technique with a non-vascularised ipsilateral fibular autograft stabilised with K-wires and a Limb Reconstruction System (LRS) external fixator. The patient demonstrated progressive clinical and radiological recovery with complete remodelling of the femur and regeneration at the fibular donor site. At final follow-up, the child had achieved painless full weight-bearing without deformity or limb length discrepancy. This case highlights the remarkable remodelling potential of bone in early childhood and suggests that minimally invasive reconstruction using a non-vascularised fibular graft may serve as an effective limb-salvage option in carefully selected paediatric patients while avoiding more complex staged reconstructive procedures.

## Introduction

Segmental bone loss of the femur following high-energy trauma represents one of the most challenging problems in orthopaedic reconstruction, particularly in the paediatric population. These injuries are often associated with open fractures, soft-tissue damage, contamination, and vascular compromise, all of which adversely affect bone healing and increase the risk of infection, nonunion, and limb dysfunction. The fundamental goals of treatment include eradication of contamination, restoration of skeletal continuity, preservation of limb length and alignment, and achievement of functional ambulation [1,2].

Several reconstructive strategies have been described for managing critical-sized femoral defects [3,4]. These include distraction osteogenesis using the Ilizarov technique, the induced membrane (Masquelet) technique, vascularised bone grafts, and non-vascularised structural grafts [5-10]. While vascularised fibular grafts provide biologically active bone with intrinsic blood supply and the potential for hypertrophy, they require microsurgical expertise and are associated with donor-site morbidity [7,8]. The induced membrane technique has also gained popularity as a staged reconstruction method, but requires multiple procedures and adequate bone graft volume [5,6,11]. Distraction osteogenesis allows gradual bone regeneration but is associated with prolonged treatment duration and external fixation-related complications [4,9,10].

In children, reconstructive challenges are further complicated by skeletal growth and the need to preserve long-term limb function. Despite these challenges, paediatric bone possesses significant regenerative and remodelling potential. However, reports describing traumatic femoral bone loss in very young children remain scarce in the literature [2,3,12].

We present a rare case of traumatic femoral segmental bone loss in a three-year-old child managed successfully using a minimally invasive technique with a non-vascularised ipsilateral fibular graft. To the best of our knowledge, this represents one of the youngest reported patients undergoing such reconstruction.

## Case presentation

**Figure 1:**
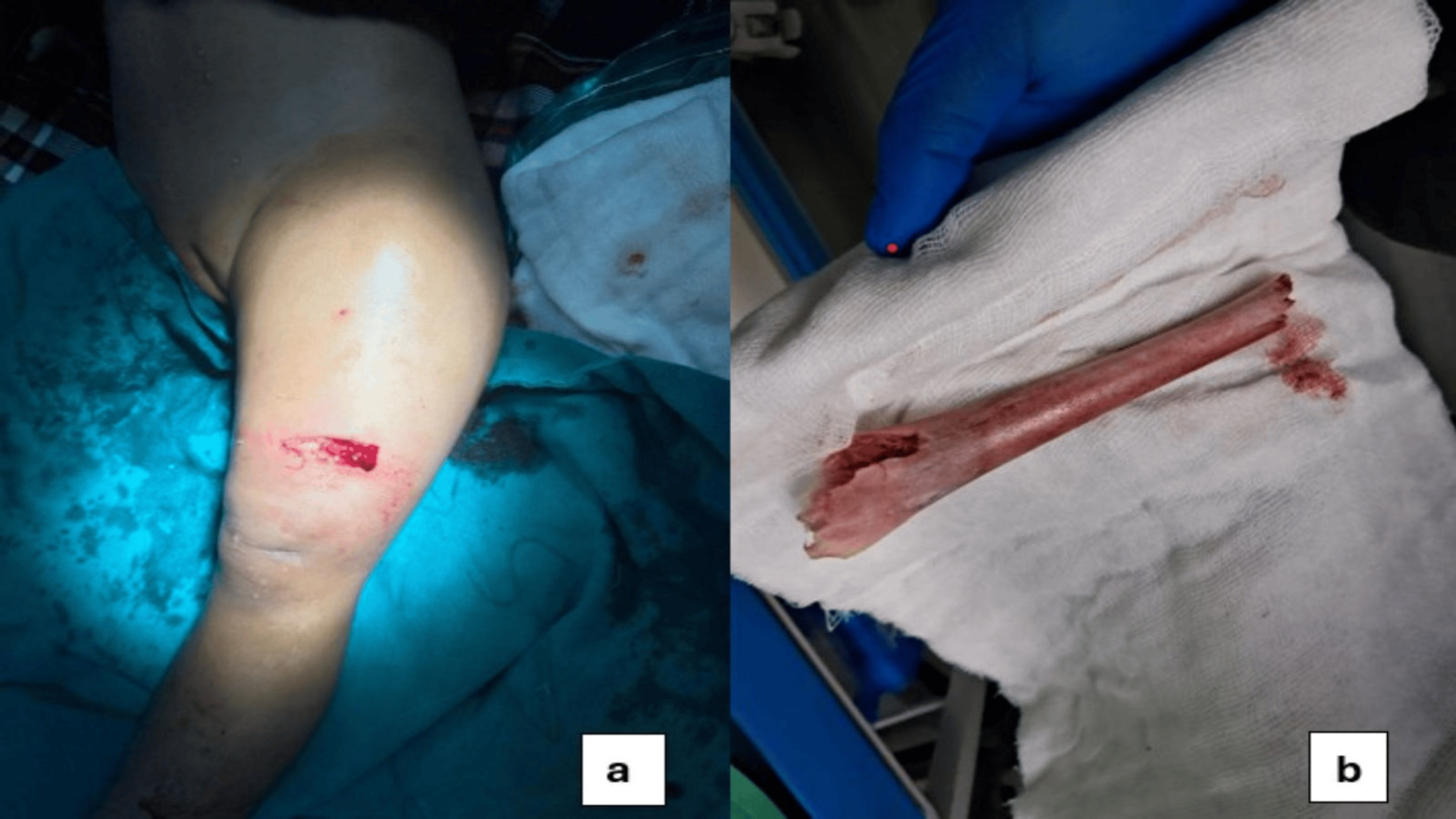
Exit wound over the distal left thigh at the time of presentation (a) and extruded femoral fragment (b).

We report the case of a three-year-old child who sustained traumatic extrusion of a segment of the femoral shaft following a motor vehicle accident. The child presented to the emergency department within 12 hours of injury with stable vital signs, minimal blood loss, and a small wound measuring approximately 4 × 1 cm near the knee. The extruded femoral fragment was brought by the attendant, wrapped in cloth (Figure 1).

**Figure 2:**
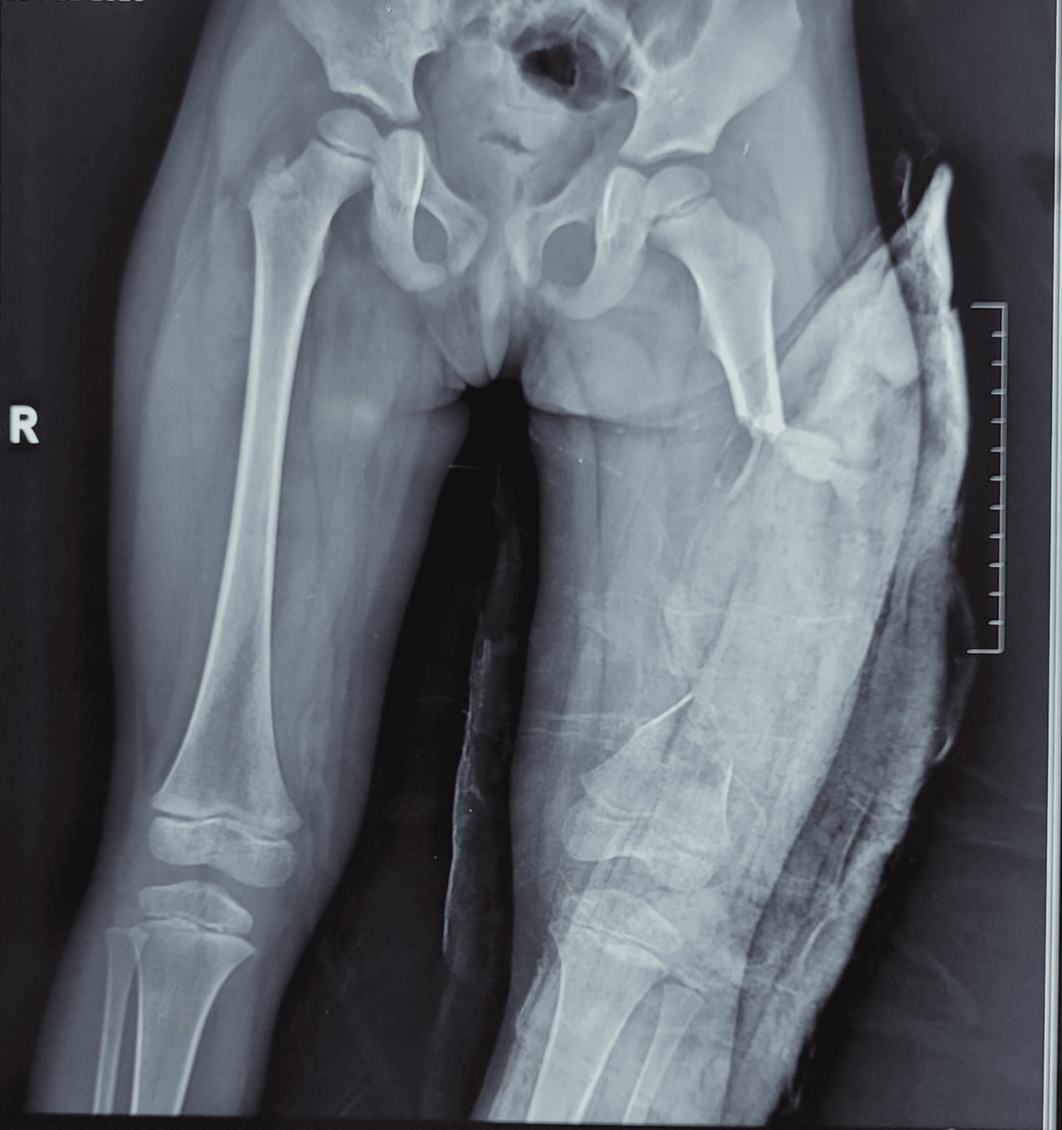
Anteroposterior (AP) view radiograph showing missing segment of the left femur (the limb was immobilised in a high groin plaster of Paris slab).

Initial management consisted of thorough wound irrigation, sterile dressing, and limb immobilisation using a plaster of Paris (POP) slab, followed by admission to the paediatric intensive care unit (Figure 2). Intravenous antibiotic coverage with a third-generation cephalosporin was initiated at presentation and continued for an additional two weeks following definitive surgery. Serial wound inspections and dressings were carried out. As the child’s general condition improved and no clinical or laboratory evidence of infection was observed, definitive reconstruction was planned after two weeks. Preoperative laboratory investigations performed prior to definitive reconstruction included a complete blood count, erythrocyte sedimentation rate (ESR), and C-reactive protein (CRP). Haemoglobin was recorded at 11 g/dL, while the total leukocyte count was 8,000 cells/mm³. The ESR was mildly elevated at 30 mm in the first hour; however, the CRP level was below 5 mg/L, with no laboratory evidence suggestive of active infection.

**Figure 3:**
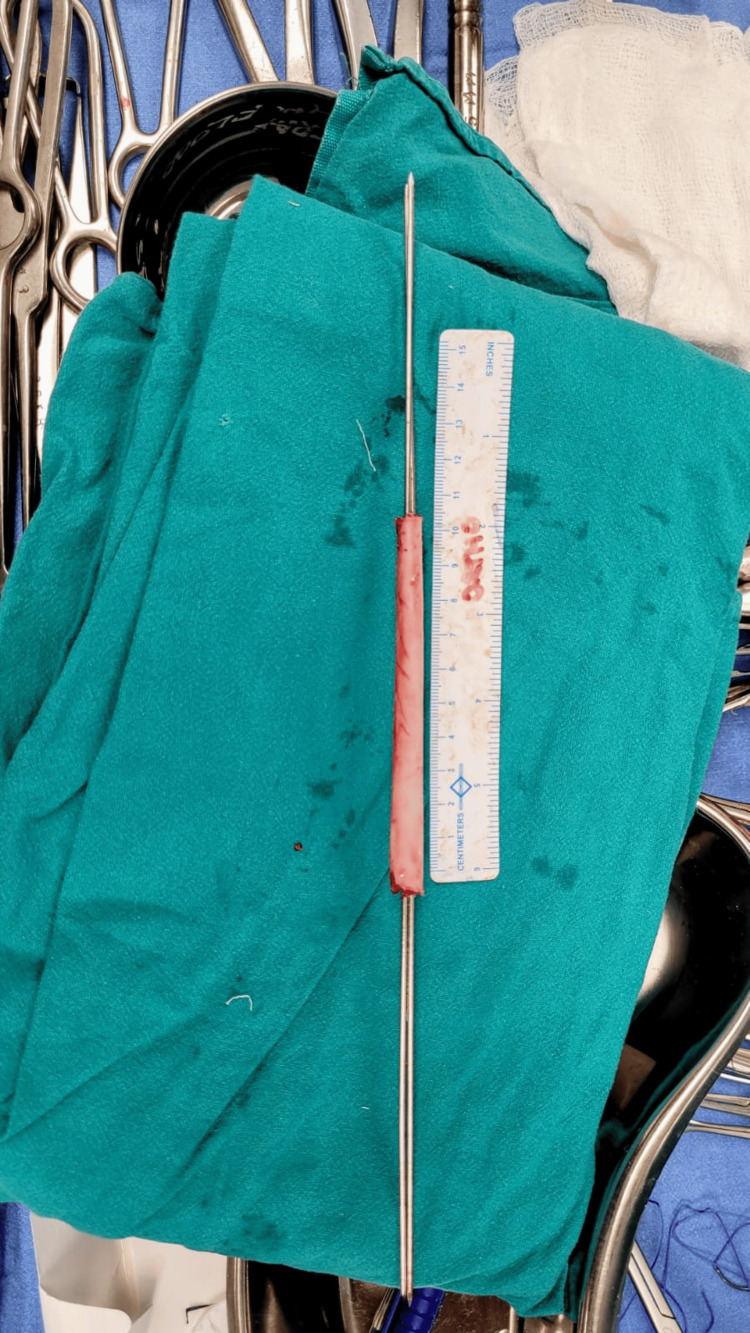
Fibular autograft harvested from the ipsilateral limb showing two K-wires (2 mm) passing through the graft for stabilisation to the femur.

Intraoperatively, under fluoroscopic guidance, the femoral defect was remeasured and confirmed to correspond to the length of the extruded fragment. A non-vascularised ipsilateral fibular autograft measuring approximately 10 mm in width and 100 mm in length was harvested through two small incisions placed at the proximal and distal third junctions of the leg (Figure 3).

**Figure 4:**
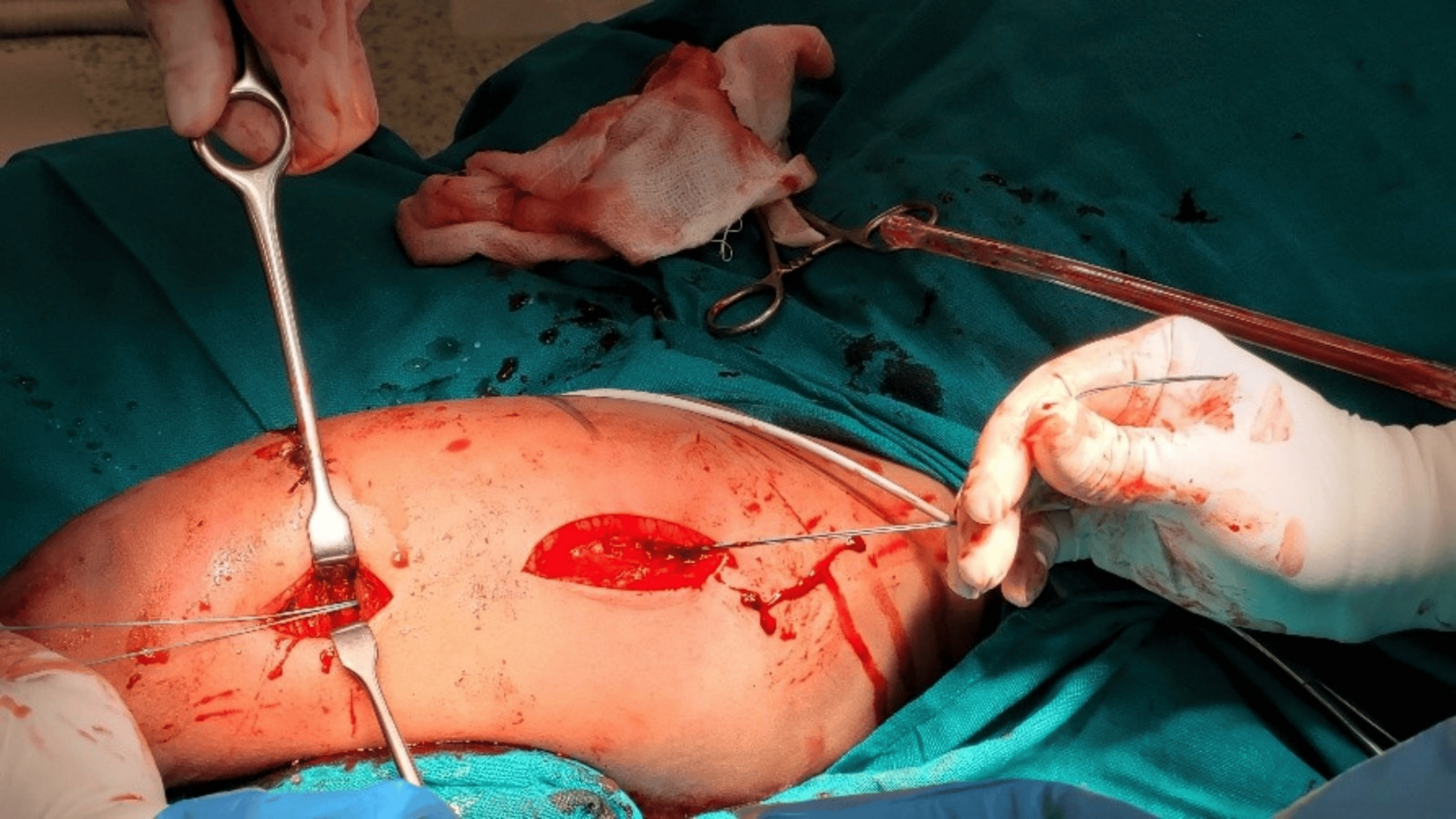
Incisions on the lateral aspect of the left thigh through which a non-absorbable suture was passed to facilitate passage of the fibular graft.

To preserve the biology of the affected limb, two limited incisions were made on the lateral aspect of the thigh, maintaining a skin bridge of approximately 80 mm and minimising soft tissue disruption. The proximal and distal femoral fragments were identified with minimal dissection. A Kelly forceps was passed from the proximal to the distal incision along the presumed tract of the extruded segment through which a non-absorbable No. 5 suture was attached and retrieved proximally to facilitate graft passage (Figure 4). The graft was then secured to the suture and gently shuttled proximally from the distal incision. Under fluoroscopic control, the graft was accurately docked into the proximal and distal femoral fragments. Intraoperatively, a wound culture sample obtained from the distal wound was sent for microbiological analysis and subsequently showed no bacterial growth.

**Figure 5:**
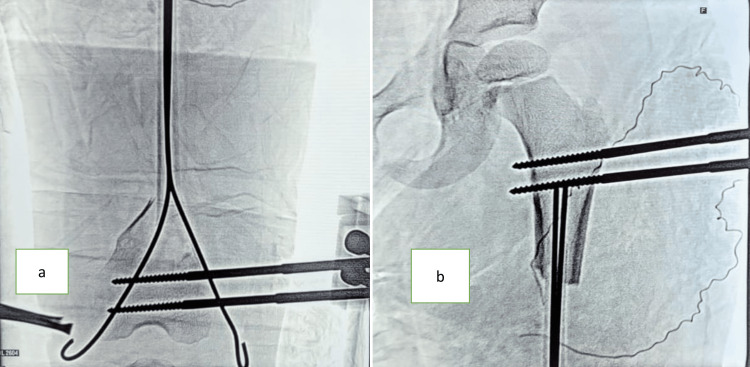
Additional stability was provided using a Limb Reconstruction System (LRS) external fixator.

Stabilisation was achieved by introducing two 2-mm K-wires through the distal femur, traversing the graft in a manner similar to titanium elastic nailing, and advancing them into the proximal fragment. Additional stability was provided using a Limb Reconstruction System (LRS) external fixator (Figure 5). Satisfactory alignment and reduction were confirmed on anteroposterior and lateral fluoroscopic views. All wounds were then closed and dressed.

**Figure 6:**
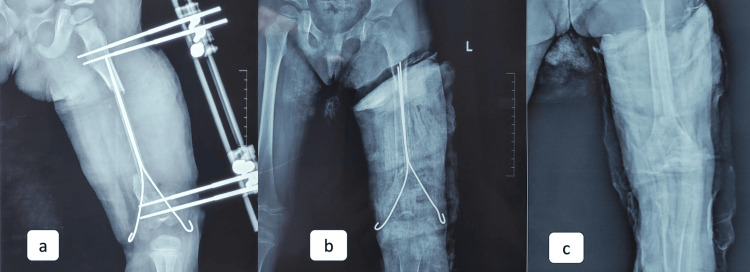
Postoperative radiograph (a) showing the fibular graft held in situ with K-wires and LRS; AP view radiograph at six weeks (b) following removal of LRS; AP view radiograph after eight weeks (c) following removal of K-wires (limb supported by a plaster of Paris slab). AP, anteroposterior; LRS, Limb Reconstruction System.

The postoperative course was uneventful. Wound dressing and pin-track care were initiated on the third postoperative day, followed by radiographic evaluation. Due to poor compliance, dressing changes were performed under sedation in the operation theatre on a weekly basis for the first six weeks. At six weeks postoperatively, radiographs demonstrated early femoral reformation. In view of this satisfactory progress, the LRS frame was removed while retaining the K-wires, and additional protection was provided using a high groin POP slab. At eight weeks, the K-wires were removed, and immobilisation was continued with an above-knee POP slab for a further four weeks. During this period, the child began full weight-bearing while in the cast, which was subsequently removed at the end of this phase (Figure 6).

**Figure 7:**
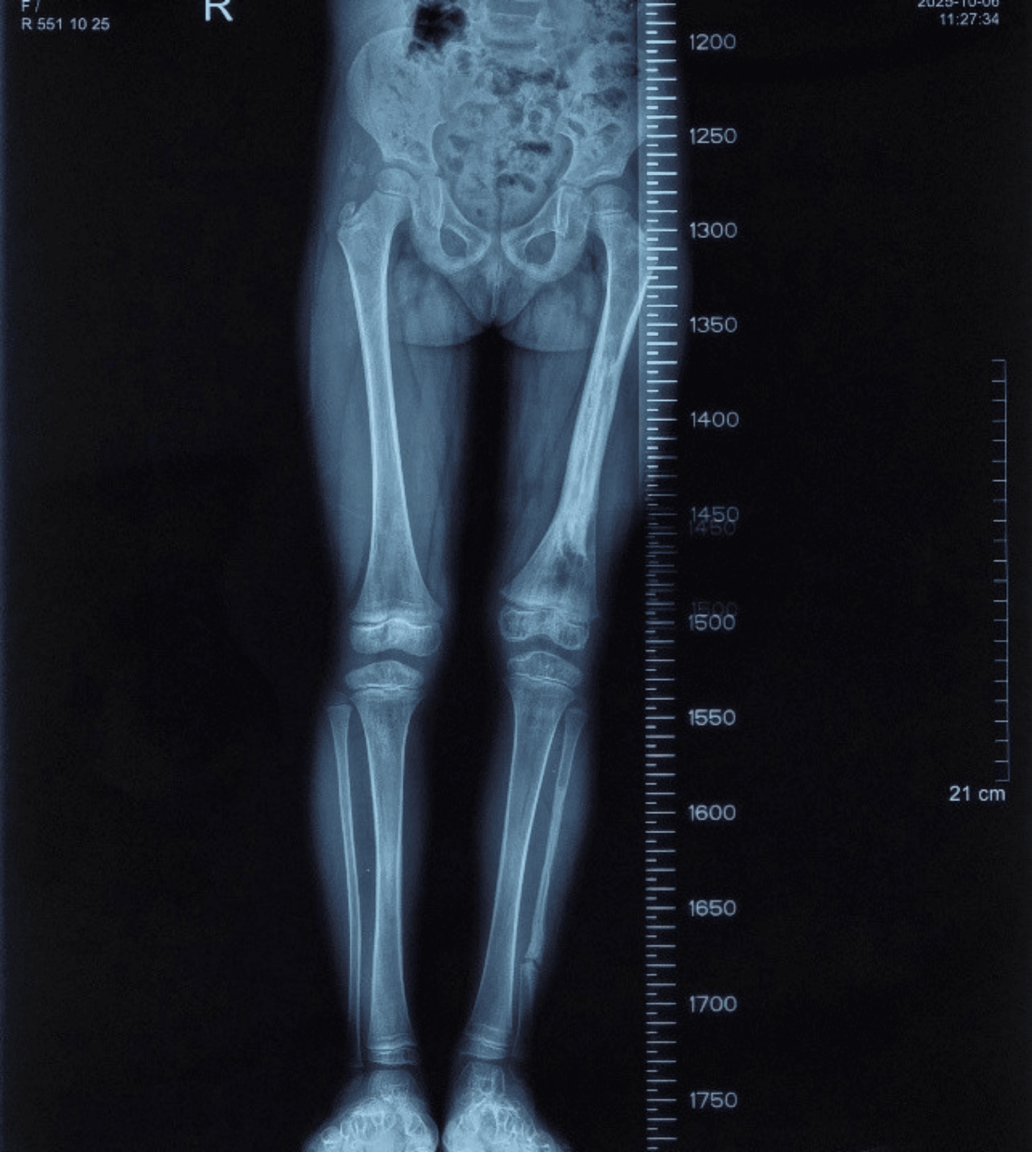
Full length anteroposterior (AP) view radiograph showing remodelling of the femur over the graft at eight months.

The patient was followed up on regularly on a monthly basis. At eight months postoperatively, radiographs demonstrated complete reformation of the femur, and the child remained asymptomatic (Figure 7).

**Figure 8:**
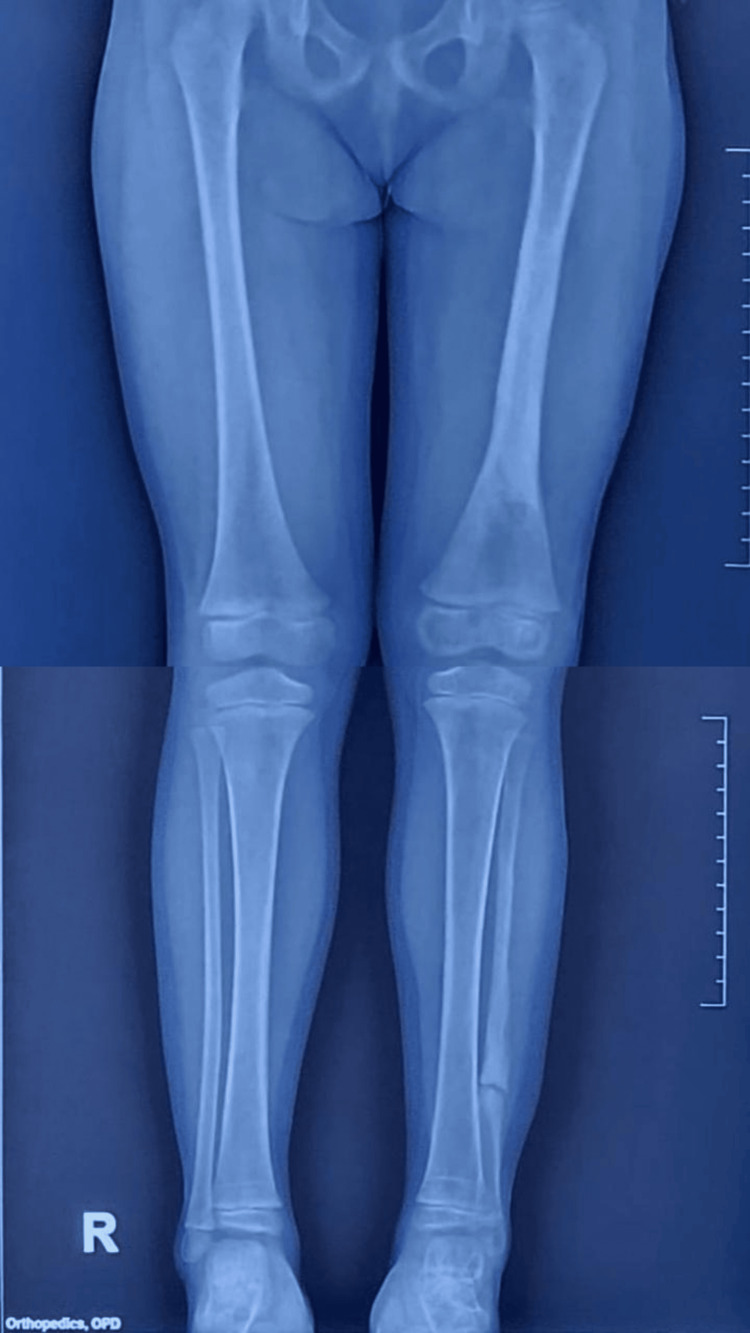
Anteroposterior (AP) view radiograph at one year showing complete remodelling of the femur, including regeneration at the site of fibular graft harvest.

At one-year follow-up, the child was able to bear full weight without difficulty and had regained a full range of motion at both the hip and knee joints. There was no limb length discrepancy, and radiographs confirmed complete femoral regeneration, including at the fibular graft donor site (Figure 8).

## Discussion

Traumatic segmental bone loss of the femur remains a formidable reconstructive challenge, particularly in the paediatric population. These injuries are frequently associated with severe soft-tissue trauma and contamination, creating an unfavourable biological environment for fracture healing. The primary objectives of treatment are restoration of limb length and alignment, achievement of stable fixation, prevention of infection, and preservation of long-term limb function [1,2].

Various reconstructive techniques have been described for the management of critical-sized femoral defects [3,4]. Distraction osteogenesis using the Ilizarov technique allows gradual bone regeneration but often requires prolonged external fixation and multiple procedures [4,9,10]. The Masquelet-induced membrane technique has also been widely used, particularly in contaminated injuries, but it requires staged reconstruction and substantial bone grafting [5,6,11]. Vascularised fibular grafts provide structural support with intrinsic vascularity and the ability to hypertrophy over time, but the procedure requires microsurgical expertise and may be associated with donor-site morbidity [7,8].

In the present case, reconstruction was performed using a non-vascularised ipsilateral fibular graft, which provided a simple yet effective biological solution for bridging the femoral defect. Non-vascularised fibular grafts offer several advantages, including shorter operative time, technical simplicity, and avoidance of complex microsurgical procedures. This approach may be particularly valuable in emergency situations or in settings where microsurgical expertise is not readily available.

One of the most remarkable aspects of this case is the age of the patient. At three years of age, this child represents one of the youngest reported cases of femoral reconstruction using a fibular graft following traumatic bone loss [2,3]. Paediatric bone has a remarkable capacity for regeneration and remodelling, which played a crucial role in the successful outcome. The complete reformation of the femur observed in this patient, including regeneration at the fibular donor site, highlights the extraordinary osteogenic potential present during early childhood.

Despite the favourable outcome, several challenges and potential risks must be considered with this technique. These include infection, graft resorption or dissolution, delayed union, and mechanical instability. Achieving adequate stability and preserving the biological environment of the fracture site are therefore critical for successful graft incorporation. In the present case, stabilisation using K-wires combined with an external fixation system allowed maintenance of alignment while minimising additional soft-tissue disruption.

An additional important aspect highlighted by this case is the role of patient and family cooperation in paediatric orthopaedic reconstruction. The prolonged treatment period, repeated dressing procedures, and need for strict follow-up required significant involvement from the child’s caregivers. Establishing trust with the family was essential for ensuring compliance with postoperative care and follow-up visits, which ultimately contributed to the successful outcome.

Given the rarity of traumatic femoral bone loss in very young children, each successfully treated case contributes valuable knowledge to the existing literature and may help guide future treatment strategies [1-3].

## Conclusions

Traumatic segmental femoral bone loss in very young children is an extremely rare and complex orthopaedic problem. This case demonstrates that reconstruction using a non-vascularised ipsilateral fibular graft, combined with stable fixation, can achieve excellent functional and radiological outcomes.

The remarkable remodelling capacity of bone in early childhood played a pivotal role in the complete regeneration of the femur observed in this patient. Compared with more complex reconstructive procedures, this technique avoids the need for multiple major surgeries and microsurgical reconstruction, while still providing effective biological and mechanical support for bone healing. This case highlights the potential of biological reconstruction using a non-vascularised fibular graft as a viable limb-salvage option in carefully selected paediatric patients with traumatic femoral bone defects.
